# The relationship between cannabis use and IVF outcome—a cohort study

**DOI:** 10.1186/s42238-021-00099-5

**Published:** 2021-09-07

**Authors:** Eden Har-Gil, Ayala Heled, Marjorie Dixon, Abdul Munaf Sultan Ahamed, Yaakov Bentov

**Affiliations:** 1 Health Sciences - Wilfrid Laurier University, Waterloo, ON Canada; 2 Health Sciences - McGill University, Montreal, QC Canada; 3 OBGYN Department, University of Toronto, Toronto, ON Canada; 4 Anova Fertility, Toronto, ON Canada; 5 OBGYN Department, McMaster University, Hamilton, ON Canada; 6 Hadassah Mount Scopus Hospital and Hebrew University, Jerusalem, Israel

**Keywords:** Cannabis, Marijuana, IVF, In vitro fertilization, Infertility, Assisted reproductive techniques

## Abstract

**Background:**

The effects of cannabis use on male and female reproduction have been the focus of scientific research for decades. Although initial studies raised concerns, more recent studies were reassuring. Considering the recent legalization of recreational use of cannabis in Canada, we sought to analyze IVF outcomes among users and non-users in a single IVF center.

**Methods:**

This is a retrospective cohort study from a single IVF center assessing IVF outcomes among male-female, non-donor IVF patients that are either cannabis users or non-users. We analyzed the ongoing pregnancy rate as well as oocyte yield, fertilization rate, peak serum estradiol, sperm, and embryo quality. We used the Mann-Whitney test, chi-square test, and Kruskal-Wallis tests where appropriate.

**Results:**

Overall, the study included 722 patients of which 68 (9.4%) were cannabis users, most defined as light users. The results of the study show similar implantation rate (40.74% vs. 41.13%) and ongoing pregnancy rate (35.2% vs. 29.1%) between the users and non-users, respectively. No significant difference between users and non-users in any of the other analyzed outcomes could be detected.

**Conclusions:**

The results may provide some reassurance for the lack of any demonstrable detrimental effects of cannabis consumption on IVF outcomes. This study was limited by its retrospective nature, self-reporting of cannabis use, and a small user sample size. A larger prospective study is needed to validate its findings.

## Introduction

Cannabis is the most used recreational drug worldwide (Maccarrone et al. 2021). Recently, its use has become even more widespread with the legalization of its medical and recreational consumption (Bayrampour and Asim 2021). The use of cannabis for recreational use is especially popular amongst men and women in the reproductive age (Volkow et al. 2019; Skelton, Hecht, and Benjamin-Neelon 2020; Beyer et al., 2019). Knowledge on the impact of cannabinoids on fertility is limited and often contradicting.

Pre-clinical studies in a rodent model have shown inhibition of spermatogenesis and decreased fertilization (Nahas et al. 2002; Dalterio et al. 1982). Furthermore, early human studies on the effects of both acute and chronic exposures to cannabis reported on concerning findings including a detrimental effect on spermatogenesis and sperm function as well as erectile dysfunction and testicular cancer (Rajanahally et al. 2019) (Schuel et al. 1994; Amoako et al. 2013; Schuel et al. 1987; Hong et al. 1982; Whan et al. 2006; Chang et al. 1993; Rossato et al. 2005).

These concerns were augmented by molecular studies showing a ubiquitous spread of the cannabinoid receptors in both male and female reproductive systems (Amoako et al. 2013; Chang et al. 1993; Rossato et al. 2005). However, while older studies of different designs expressed an almost unanimous concern over adverse effects of cannabis use to both male and female reproductive outcomes, more recent studies are challenging this paradigm by showing a similar reproductive outcome for cannabis users of both genders (Kasman et al. 2018).

As data on the effects of cannabis exposure on male and female fertility as well as information on the effects on IVF outcome is contradicting, there is no clear answer to whether the current use of cannabis in either men or women affects their reproductive function.

Since the legalization of recreational cannabis use in Canada and large parts of the USA has resulted in increased access and popularity of its use, we sought to examine whether there is a difference in IVF outcome between all cannabis users and non-users in a single IVF clinic.

## Purpose

The aim of this single clinic-based cohort retrospective study is to compare the outcome of IVF treatment among cannabis users vs. non-users. The main outcome measure we choose was the ongoing pregnancy rate. Secondary outcomes are detailed in the “Methods” section.

## Methods

The study was approved by the McMaster University (Hamilton Integrated Research Ethics Board)—project #5024.

The study is a retrospective cohort study that included all patients that completed oocyte retrieval and embryo transfer at the ANOVA IVF center since its initiation in September 2016 and until September 2019. To be eligible, charts had to contain information on cannabis use status.

Patients referred to the ANOVA IVF center are routinely asked during the initial visit to fill a questionnaire that includes questions on the use of recreational drugs including the type and frequency for both partners. Since we intended to assess the effects of cannabis use on male and female reproduction, we did not include same-sex couples, couples using donor oocytes or donor sperm, and couples using a gestational carrier. Based on the information provided on the use of cannabis by each couple, they were allocated to a group of non-users or users. We also did a subgroup analysis based on the identity of the user: female, male, or both. For the main outcome measure, we had also analyzed the intensity of cannabis use (light—up to 3 times a week; heavy—more than 3 times a week).

Charts for all eligible patients for screening for the following outcomes:

1. Ongoing pregnancy rate (percentage of cycles that resulted in a pregnancy that was still ongoing at the time of patient discharge from the clinic by the end of the first trimester) and implantation rate (the number of intrauterine gestational sacs divided by the number of embryos that were transferred to the uterus)—primary outcome.
2. Oocyte (mature) yield—the number of oocyte (mature) yield—the percentage of oocytes (total or mature) aspirated from the total number of mature ovarian follicles (14–25 mm in diameter) as measured on the day in which ovulation was triggered.
3. Peak serum estradiol.
4. Fertilization rate—the percentage of oocytes that were fertilized out of all oocytes that were inseminated. This rate was divided into fertilization rate with intracytoplasmic sperm injection (ICSI) vs. standard insemination.
5. Sperm quality (normal/fair/poor)—based on the index detailed below.
6. Blastocyst formation rate—the percentage of embryos that developed into a blastocyst out of all fertilized oocytes (zygotes).
7. High-quality blastocyst rate (number of blastocysts graded 3–6 AA/AB/BA out of all normally fertilized zygotes (2PN))—this rate reflects the development of top-quality embryos after 5–6 days in culture according to the Schoolcraft-Gardiner grading system out of the total number of normally fertilized oocytes (Gardner and Schoolcraft 1998).

We had also reviewed the embryology reports for any written comments on any unusual features observed by the embryologist during oocyte, sperm, or embryo examination.

For the analysis of sperm quality, we employed the following grading system that defined the sperm as being either normal, fair, or poor based on the following criteria:

**Table:** 

Sperm concentration:	Total motility:	Normal form:
1: < 15 M/ml	1: < 30%	1: < 4%
2: 15–25 M/ml	2: 30–50%	2: 4–10%
3: > 25 M/ml	3: > 50%	3: > 10%

Scores from all three categories were summed. Sperm was regarded as poor, fair, or normal if the total score was 1–3, 4–6, or 7–9, respectively.

## Statistical analysis

The data was tested for normality of distribution using the Anderson-Darling test, the D’Agostino, Pearson test, and the Shapiro-Wilk test.

As the data was not found to be normally distributed, we used a non-parametric test (Mann-Whitney test) for all continuous parameters combined. Categorical variables were analyzed with the chi-square test.

We used the Kruskal-Wallis test for comparison between multiple groups for the subgroup analysis.

A power analysis was done using the G*Power software version 3.1.9.2 (Axel Buchner - University of Dusseldorf, Edgar Erdfelder - University of Mannheim, Franz Faul - University of Kiel, and Albert-Georg Lang - University of Dusseldorf). According to the following parameters: Wilcoxon-Mann-Whitney test (two groups) one tail, effect size 0.5, an err probability of 0.05, power 0.95, and allocation ratio N2/N1 1, the calculated sample size was 92 in each arm.

The statistical analysis was performed using GraphPad Prism version 9.0.1 for Windows, GraphPad Software, San Diego, CA, USA, www.graphpad.com.

## Results

The study population consisted of 722 patients that completed oocyte retrieval and embryo transfer at the ANOVA IVF center. Of these patients, there were 654 non-users (study group 1) and 68 (9.4%) couples in which either the patient, partner, or both reported on cannabis use (study groups 2–9). In most user couples, either the female or male partners were cannabis users (57%), and in most couples, the level of use of either partner or both was defined as mild (65%). There were 15 couples in which the female partner was a cannabis user, forty couples in which the male partner was a cannabis user, and 13 couples in which both partners were using cannabis. Due to the small numbers of patients included in the user subgroups, we conducted the comparison with all users pulled into one group as well as a subgroup analysis according to the identity of the user. Patient demographics are described in Table 1: The median patient age of users was significantly younger than non-users (34 ± 4.1 vs. 36 ± 4.4; *P* value = 0.012); however, ovarian reserve for the two groups as estimated by serum anti-Mullerian hormone (AMH) was similar (user group 16.5 ± 14.2 pmol/L vs. non-user group 15.5 ± 22.6 pmol/L; *P* value 0.90).

**Table 1:** Baseline demographic characteristics of 722 couples seeking IVF: median, standard deviation, missing data (MD), *P* value describing the difference between cannabis users and non-users including a subgroup analysis of the user group according to the identity of the user (female partner, male partner, or both). The data relates to the patient’s age and serum anti-Mullerian hormone (AMH) that represents the ovarian reserve at the time the IVF treatment was conducted

	Users vs. non-users				Subgroup analysis			
	Normal distribution^**a**^	Non-users, mean/median (SD)	Users, mean/median (SD)	***P*** value^**b**^	Female users, mean/median (SD)	Male users, mean/median (SD)	Both users, mean/median (SD)	***P*** value^**c**^
*Age*	No	35.4/36 (4.4)	34.2/34 (4.1)	0.012	34.1/34 (4.4)	34.2/34 (4.1)	34.4/34 (3.4)	0.11
	MD	0	0		0	0	0	
*AMH*	No	22.5/15.5 (22.6)	19.2/16.5 (14.2)	0.900	23.5/18.2 (22.7)	18.2/15.6 (10.5)	17.5/19.6 (11)	0.985
	MD	91 (13.9%)	10 (14.7%)		3 (14.7)	7 (17.5)	0	

^a^The data was tested for normality of distribution using the Anderson-Darling test, D’Agostino and Pearson test, and Shapiro-Wilk test.

^b^Comparison between users and non-users was conducted with the Mann-Whitney test

^c^The subgroup analysis was done using the Kruskal-Wallis test

Reproductive outcomes compared between cannabis users and non-users included several parameters that are detailed in Tables 2 and 3. The outcome parameters that represent the response of the ovaries to ovarian stimulation that were analyzed included the number of mature follicles (> 15 mm), peak serum estradiol, and the number of oocytes. These were shown to be similar between users and non-users. Parameters that represent the response of the ovary to the hCG trigger: the number of mature oocytes (MII), oocyte yield (number of oocytes divided by the number of mature follicles), and mature oocyte yield (number of mature oocytes out the number of mature follicles), were also found to be similar between the groups. We had also examined the potential effects of cannabis use on sperm quality looking at the volume of the semen, sperm progressive motility, and a composite index described earlier, grading sperm concentration, motility, and morphology. There were no significant differences in any of the sperm parameters between the group of users and non-users. We analyzed the parameters that relate to the process of fertilization including oocyte fertilization by standard IVF (co-incubation of sperm and oocytes) or via injection of a single sperm into the cytoplasm of the oocyte (ICSI). We were not able to detect any significant differences in these parameters between users and non-users. We went on to analyze the outcome parameters that represent early embryonic development. We calculated the percentage of embryos that developed into a blastocyst (the stage of embryo development that precedes implantation achieved typically on days 5–6 of embryo culture). We also compared the rate of top-quality blastocyst development between the two groups. Neither one of the early embryonic development parameters differed between the groups.

**Table 2:** IVF treatment characteristics of 722 IVF patients divided based on the level of cannabis use—mean, median, standard deviation, missing data, *P* value, and test used to compare the difference between cannabis users and non-users including a subgroup analysis of the user group according to the identity of the user (female partner, male partner, or both). Abbreviations: *MII* meiosis two oocyte-mature oocytes, *2PN* 2 pronuclei oocyte/zygote/normally fertilized oocyte, *ICSI* intracytoplasmic sperm injection (a method for oocyte fertilization with a single sperm injected into its cytoplasm), *IVF* in vitro fertilization with insemination of oocytes with exposure to motile sperm as opposed to ICSI, *Blsts* blastocysts (the pre-implantation stage of embryo development), *HQ* high-quality. The left columns describe the comparison of users and non-users, and the columns on the right describe the comparison of users based on the gender of the user

Outcome measure	Users vs. non-users					Subgroup analysis				
	Normal distribution	Non-users, mean/median (SD)	Users, mean/median (SD)	***P*** value	Test used	Female users, mean/median (SD)	Male users, mean/median (SD)	Both users, mean/median (SD)	***P*** value	Test used
*Number of mature follicles*	No	10.5/9.0 (6.3)	10.5/11 (6.0)	0.538	Mann-Whitney	13/12 (7.7)	9.95/10 (5.2)	9.31/8 (5.1)	0.513	Kruskal-Wallis
	MD	29 (4.44%)	0			0	0	0		
*Number of oocytes*	No	12.65/11 (8.9)	12.79/12.5 (6.6)	0.998	Mann-Whitney	13.1/13 (6.7)	13.5/12.5 (7.0)	10.23/10 (4.3)	0.350	Kruskal-Wallis
	MD	8 (1.23%)	0			0	0	0		
*Number of MII oocytes*	No	9.6/8.0 (6.7)	9.3/8.5 (5.0)	0.909	Mann-Whitney	9.5/9 (5.2)	9.8/9 (5.2)	7.7/8 (3.5)	0.756	Kruskal-Wallis
	MD	15 (2.3%)	0			0	0	0		
*Oocyte yield (%)*	No	130/114 (84) (84(83%)	139/120 (95)	0.423	Mann-Whitney	111/100 (38)	154/128 (116)	123/114 (56)	0.433	Kruskal-Wallis
	MD	32 (4.9%)	0			0	0	0		
*Mature oocyte yield (%)*	No	98/98 (64)	100/100 (51)	0.723	Mann-Whitney	86/75 (43)	107/96 (54)	95/93 (47)	0.597	Kruskal-Wallis
	MD	43 (6.58%)	0			0	0	0		
*Peak estradiol*	No	11,882/9863 (8287)	11,309/10,016 (6445)	0.950	Mann-Whitney	11,886/10,115 (7061)	11,173/9926 (6266)	11,065/10,057 (6199)	0.988	Kruskal-Wallis
	MD	6 (0.92%)	0			0	0	0		
*Sperm volume (ml)*	No	2.76/2.55 (1.9)	2.65/2.5 (1.6)	0.563	Mann-Whitney	***1.77/1.6 (1.16)***	2.69/2.5 (1.6)	***3.53/4.2 (1.3)***	0.013	Kruskal-Wallis
	MD	22 (3.37%)	0			0	0	0		
*Progressive motility (%)*	No	18/15 (14)	20.7/17 (14)	0.1.51	Mann-Whitney	18/11 (14)	21/15 (13.5)	23/20 (15)	0.326	Kruskal-Wallis
	MD	86 (13.2%)	5 (7.5%)			2 (13%)	3 (7.5%)	0		
*Sperm quality*	No	6 (2.2)	6 (1.9)	0.500	Mann-Whitney	***5 (1.4)***	***6 (1.4)***	6 (2.2)	0.022	Kruskal-Wallis
	MD	20 (3.6%)	0			0	0	0		
*Number of 2PN*	No	7.5/6.0 (5.5)	7.1/7.0 (4.0)	0.785	Mann-Whitney	7.9/8 (3.5)	7.1/7 (4.3)	6.3/7 (3.3)	0.785	Kruskal-Wallis
	MD	14 (2.18%)	0			0	0	0		
*ICSI fertilization rate (%)*	No	81/83 (19.4)	79/85 (22.7)	0.977	Mann-Whitney	86/87.5 (14.5)	78/85 (24)	77/84.6 (25)	0.860	Kruskal-Wallis
	MD	66 (3.06%)	2 (2.94%)			0	1 (2.5%)	1 (7.7%)		
*IVF fertilization rate (*%)	No	71/75 (25.4)	62/75 (33)	0.407	Mann-Whitney	89/87 (8)	53/53 (37)	63/75 (16)	0.238	Kruskal-Wallis
	MD	548 (83.9%)	54 (79.4%)			12 (80%)	30 (75%)	10 (77%)		
*Number of blasts on day 5/6*	No	4.1/3.0 (3.9)	3.8/3.0 (2.6)	0.7133	Mann-Whitney	3.7/3.0 (2.24)	4.1/3.5 (2.8)	2.9/3 (1.6)	0.801	Kruskal-Wallis
	MD	131 (20.6%)	7 (10.3%)			1 (6.6%)	5 (12.5%)	1 (7.7%)		
*Number of top-quality blasts*	No	2.0/1.0 (2.6)	1.6/1.0 (1.6)	0.854	Mann-Whitney	1.6/1.5 (1.2)	1.67/1 (1.79)	1.6/1 (1.49)	0.973	Kruskal-Wallis
	MD	122 (18.6%)	6 (8.8%)			1 (6.6%)	4 (10%)	1 (7.7%)		
*Blastocyst formation rate (%)*	No	49/50 (33)	55/55 (29.6)	0.289	Mann-Whitney	51/50 (26)	58/59 (32)	51/40 (24)	0.563	Kruskal-Wallis
	MD	23 (3.5%)	2 (2.9%)			0	1 (2.5%)	1 (7.7%)		
*HQ blastocyst formation rate (%)*	No	24/17 (25)	24/17 (24.7)	0.897	Mann-Whitney	27/23.6 (24.6)	20/14.8 (21.2)	29/16.7 (32)	0.791	Kruskal-Wallis
	MD	124 (18.9%)	6 (8.8%)			1 (6.6%)	4 (10%)	1 (7.7%)

**Table 3:** Treatment outcome for 722 IVF patients based on the level of cannabis use: implantation rate (IR) and ongoing pregnancy rate (OPR) among the different study groups as well as combining all users. The symbols in the first two rows define the level of use for the two partners: 0, no use; +, light use; ++, heavy use

Group	Level of use								All users	
	G1	G2	G3	G4	G5	G6	G7	G8		
**Female**	0	+	++	+	++	0	0	+		
**Male**	0	0	0	+	++	+	++	++		
**Number**	654	13	2	5	4	26	14	4	68	*P* value
**Implantation rate per embryo transfer**	41.13%	53.85%	0	37.5%	50%	47.06%	27.8%	0	40.74%	0.45
**Ongoing pregnancy rate per cycle started**	29.10%	43.75%	0	42.86%	40.00%	40.0%	25.0%	0	35.23%	0.508

describes the rate of implantation per embryo transfer as well as the ongoing pregnancy rate per ovum pickup (OPU) among the different test groups: G1, non-users; G2, female light user partner non-user; G3, female heavy user partner non-user; G4, both female and male partners are light users; G5, both female and male partners are heavy users; G6, female non-user, male light user; G7, female non-user, male heavy user; and G8, female light user and male heavy user. No significant difference in either implantation or ongoing pregnancy rate was detected using the Kruskal-Wallis test

The subgroup analysis according to the identity of the user (female, male, or both) showed no significant differences other than in sperm volume that was highest in the group in which both partners were cannabis users and in the sperm quality that was defined as highest with the male partner consuming cannabis. With regard to the primary outcome measures, implantation, and ongoing pregnancy rates, we did study the differences in the user vs. non-user groups as well as in the user subgroups (Table 3). The implantation rate (IR) per transfer for the non-user group was 41.1% and 40.7% for the users. The ongoing pregnancy (OPR) per cycle start rate was 29.1% for non-users and 35.2% for the users. The difference between the users and non-users for both the IR and OPR as well as the difference between the subgroups of users was not statistically different.

Analysis of the written comments made by embryologists analyzing the sperm, oocytes, and embryos of all the patients included in the study showed no remarkable differences between users and non-users.

## Discussion

The study presented in this paper is a retrospective cohort study that assessed multiple male and female reproductive outcome measures among all couples completing oocyte retrieval in one IVF center. As the data was collected during years in which the recreational use of cannabis was considered both legally and socially acceptable, patients faced fewer barriers to voluntarily disclose using cannabis. The prevalence of cannabis users in our study (9.4%), male users (7.3%), and female users (4%) was lower than previously reported among couples trying to conceive (Kasman et al. 2018); however, this study included only infertile patients that may be less likely to engage in a potentially unhealthy lifestyle. This rate of cannabis use is similar to the rate reported by a recent Canadian survey (Keethakumar et al. 2021).

All the reproductive outcomes of cannabis users and non-users in our study were comparable. These parameters included measures of ovarian response, sperm quality, efficiency of fertilization, early embryonic development, and implantation. In fact, the ongoing pregnancy rate per cycle start trended higher for the group of cannabis users (35.2% vs. 29.1%). This could partially relate to the female participants in the user group being younger than the non-user counterparts.

The use of cannabis is growing rapidly and gaining widespread legal and social acceptance, while the consumption of tobacco is on a continuous decline because of health concerns, legislation, and social trends (Gagne 2017). According to the Canadian Alcohol and Drug Use Monitoring Survey sponsored by Health Canada, 41.5% of Canadians aged 15 years and older have used cannabis in their lifetime and 10.2% have used cannabis in the past year alone (Porath et al., 2019). In Canada, the use of cannabis for both medicinal and recreational uses was legalized in October 2018. The legalization of cannabis is leading to an inevitable increase in its popularity especially among men and women of reproductive age. A similar trend was also evidenced among couples trying to conceive and pregnant women (Volkow et al. 2019). A recent study compared the preconception, prenatal, and post-natal prevalence use of cannabis in states that legalized recreational cannabis use versus states that did not (Skelton, Hecht, and Benjamin-Neelon 2020). The survey showed women residing in states that legalized recreational cannabis were significantly more likely to use it before and during pregnancy.

Furthermore, the widespread consumption of cannabis among males in the reproductive age raised health concerns.

The increase in the use of cannabis took place despite concerning reports from both animal and human studies associating chronic exposure to inhaled or injected cannabis with sperm abnormalities as well as the development of testicular lesions. Injection of the cannabis-derived tetra-hydro-cannabinol (THC)—the active component of cannabis—to mice was associated with the arrest of spermatogenesis (Dalterio et al. 1982; Nahas et al. 2002).

These reports followed earlier publications reporting that either in vitro or in vivo acute exposure of spermatozoa in a number of species, including humans, to several types of cannabinoids led to a reduced fertilization rate. This finding was attributed to the inhibition of the acrosome reaction as well as decreased sperm motility (Schuel et al. 1994; Amoako et al. 2013; Schuel et al. 1987; Hong et al. 1982; Whan et al. 2006; Chang et al. 1993; Rossato et al. 2005).

A large human cohort study found that a routine use of cannabis at a rate of twice a week or more was associated with a 30% reduction in sperm concentration (Gundersen et al. 2015). This rate was similar to the rate of cannabis use reported by most of the cannabis users in our study.

A recently published systematic review summarized all the in vivo and in vitro studies that assessed the effect of cannabis exposure on male infertility. The authors concluded that the use of cannabis may be associated with a reduction in sperm quality, erectile dysfunction, and testicular cancer (Rajanahally et al. 2019).

In contrast, Kasman et al. who surveyed the association between male and female cannabis use and time to pregnancy among 758 males and 1076 females that were actively trying to conceive between the years 2002 and 2015 were not able to detect any difference in the length of the time to pregnancy between either male or female cannabis users and non-users regardless of the frequency of use (Kasman et al. 2018).

In our study, we were not able to detect any differences in sperm quality parameters between cannabis users and non-users. Interestingly, sperm in the groups of male cannabis users showed the highest semen volume and the best sperm quality. A similar finding was reported by Nassan et al., in a recent report (Nassan et al., 2019a, b).

The effects of cannabis exposure on in vitro fertilization outcomes were assessed in several studies. These studies demonstrated that cannabis use is associated with a lower yield of harvested oocytes during in vitro fertilization. Furthermore, the addition of synthetic (CP 55940, WIN 55212–2), natural (THC), or endogenous (AEA and 2-AG) cannabinoids to embryo culture media resulted in arrested development of two-cell embryos as well as reduction of the number of trophectoderm cells in those blastocysts that were able to escape the developmental arrest. (Wang, Dey, and Maccarrone 2006).

A more recent study that examined the chronic exposure of male mice to THC via intraperitoneal (IP) injections that began at puberty and continued for 1 month found that despite a remarkably high testicular THC concentration, there was no significant difference in testicular size, rate of spermatogenesis, apoptosis, sperm concentration, and motility. They also compared the outcome of IVF using sperm from mice treated with THC vs. control and found no difference in the rate of fertilization and blastocyst formation (Lopez-Cardona et al. 2018). A recent study by Nassan et al. reported that couples undergoing IVF treatment in which the male partner was a current cannabis user had a significantly higher adjusted probability for a live birth (Nassan et al., 2019a, b).

The results of this study are in line with the newer studies suggesting that the use of cannabis is not associated with a compromised outcome for couples undergoing IVF. There may be several explanations for the discrepancy between the results of the newer studies on the effects of cannabis consumption on reproductive health, including this study, and the older ones. Studies that were done before the legalization of cannabis involved consumers of an illegal substance produced in a non-regulated process. Moreover, it was shown that individuals that consume illicit drugs are more likely to engage in other unhealthy behaviors (Keethakumar et al. 2021).

### Strengths and limitations of the study

This is a cohort study that compared the outcome of IVF treatments of patients originating from a single IVF clinic treated by the same clinical staff and embryology lab. The data is recent, therefore reflecting current IVF success rates. To our knowledge, this is the first study that was done after the legalization of recreational cannabis use and therefore may provide a more realistic prevalence of cannabis use among patients. One limitation of this study is that it is a retrospective study, relying on patients voluntarily reporting their cannabis use and frequency of use. This means that a portion of patients classified as “non-users” could have potentially been cannabis users, thus impacting the results of the study. Also, we did not include information on other lifestyle confounders such as tobacco use, although the rate of daily tobacco use in Canada for the duration of the study was less than 10%. Also, we were not able to record data on cannabis use during pregnancy for participants in the study. Furthermore, as our cohort included 654 non-users and 68 users, our sample was underpowered on the users’ arm.

## Conclusions

Our study did not show any detrimental impact of current cannabis use on any of the measured IVF outcomes. These results should be validated by a larger prospective study.

## Data Availability

Raw data is available upon request.

## Abbreviations

IVF: In vitro fertilization
USA: United States of America
THC: Tetra-hydro-cannabinol
LH: Luteinizing hormone
IP: Intraperitoneal
2PN: 2 Pronuclei—normally fertilized zygote
AMH: Anti-Mullerian hormone
MII: Mature oocyte
ICSI: Intracytoplasmic sperm injection
IR: Implantation rate
OPR: Ongoing pregnancy rate
OPU: Ovum pickup—aspiration of ovarian follicles to retrieve oocytes
